# Ovariotomy

**Published:** 1878-04-20

**Authors:** 


### OVARIOTOMY.

In the course of some clinical remarks on ovariotomy, made at the Samaritan Hospital, Mr. Spencer Wells detailed the great improvements that had taken place during the last few years in the diagnosis and treatment of ovarian tumors. The operation is now conducted in a light, airy, and quiet room, in which the patient remains alone with her nurse for at least a week after the operation. No visitor is admitted to witness the operation without declaring that he has attended no post mortem nor any case of infectious disease for a week previously. The patient is placed upon a table, lying on her back, warmly clothed, the lower limbs covered with a blanket, the head and shoulders supported by pillows, the knees and hands secured by straps, a perforated india-rubber sheet, so applied that only the front of the abdomen is uncovered, and she is asleep under the influence of what Mr. Wells believes to be the safest and best of known anæsthetics, bi-chloride of methylene. All the instruments that can be wanted for the most complicated case are ready and at hand. There must not be, he says, any threading of needles at the last moment. The nurses have a precise number of perfectly pure and soft sponges, and plenty of small fine linen cloths for use before the sponges are wanted. Supposing daylight direct or reflected fails, Mr. Wells has tried various kinds of reflecting lamps when searching for vessels deep in the pelvis; but the most useful of all is the small medical lamp recently introduced by Colin, of Paris. All this is ready before visitors come into the room, and they are then requested to observe the most absolute silence. It is quite a common thing for an operation to be completed without a single word having been spoken by the surgeon, the assistants or the nurses; and if a remark is made by an unwary visitor, it is at once hushed. The incision in the abdominal wall, the stopping of bleeding from superficial vessels by torsion forceps, the division of the peritoneum, the exposure and tapping of the cyst, the separation of adhesions, the management of adhering omentum or intestine, the breaking down of inner septa, and the withdrawal of the tumor from the abdominal cavity, the treatment of the pedicle, the examination of the opposite ovary and uterus, the thorough cleansing of the pelvic and peritoneal cavities, the use of drainage-tubes if required, the closing of the wound, the dressing and bandage—all are matters of detail of great importance. The patient is carried from the operation table and placed in a warm, dry bed. The room is at once cleared and darkened, and when she awakes she finds herself alone with her nurse. Recent changes in the management of the patient after operation have been chiefly in the direction of regulating temperature. Enough opium is given to relieve pain, but no more. The patient is kept warm enough to encourage free action of the skin without being made uncomfortably hot. Food and drink are regulated by the instinctive desire for them. All the nurses are instructed in the use of the thermometer, and they are directed whenever the temperature rises above 100° Fahr. to keep the head cool by means of the ice-water cap. If the skin is dry, very small doses of aconite are given frequently (half a drop of the tincture every half hour.) It is only in the rare exceptional cases of septicæmia, septic peritonitis, or pyæmic fever that large doses of quinine or of salicylate of soda are thought of. In some few cases bleeding from the arm has been necessary, but, as a rule, the patients are let alone after the operation, and they get well.—*London Practitioner*.

